# A needs assessment study for optimising prescribing practice in secondary care junior doctors: the Antibiotic Prescribing Education among Doctors (APED)

**DOI:** 10.1186/s12879-016-1800-z

**Published:** 2016-08-30

**Authors:** Myriam Gharbi, Luke S. P. Moore, Enrique Castro-Sánchez, Elpiniki Spanoudaki, Charlotte Grady, Alison H. Holmes, Lydia N. Drumright

**Affiliations:** 1 NIHR Health Protection Research Unit in Healthcare Associated Infections and Antimicrobial Resistance at Imperial College London, Hammersmith Campus, Du Cane Road, London, W12 0HS UK; 2 National Centre for Infection Prevention and Management, Hammersmith Campus, Du Cane Road, London, W12 0HS UK; 3 Imperial College Healthcare NHS Trust, Hammersmith Hospital, Du Cane Road, London, W12 0NN UK; 4 Department of Medicine, University of Cambridge, Cambridge, CB2 0QQ UK

**Keywords:** Antimicrobials, Continuing medical education, Clinical education, Knowledge, Behaviour

## Abstract

**Background:**

Appropriate antimicrobial prescribing is essential for patient care, yet up to half of antimicrobial prescriptions written in the UK are sub-optimal. Improving prescriber education has recently been promoted as a mechanism to optimise antimicrobial use, but identification of key learning objectives to facilitate this is so far lacking. Using qualitative methods we investigated junior doctor knowledge, attitudes, and behaviours around antimicrobial prescribing to identify key areas to address in future educational programmes.

**Methods:**

A cross-sectional survey of qualified doctors in training in West London was undertaken exploring antimicrobial prescribing practices and educational needs.

**Results:**

Among 140 junior doctors from 5 London hospitals, a third (34 %) reported prescribing primarily unsupervised, and two thirds (67 %) reported difficulties obtaining prescribing support outside of hours. 20 % stated not feeling confident in writing an antimicrobial prescription, but confidence was increased through having confirmatory diagnostic results (24) and obtaining advice from a senior doctor (26 %); whether this senior was from their own specialty, or an infection-specialist, varied significantly (*p* < 0.01) by experience. Only a small percentage (5–13 %; depending on number of years post-qualification) of participants stated their previous antimicrobial education was effective. 60 % of those in their first year post qualification reported wanting further education in antimicrobial prescribing, rising to 74 % among more experienced junior doctors. Specific areas of educational need identified were (i) principles of antimicrobial prescribing, (ii) diagnosis of infections, (iii) clinical review of patients with infections, (iv) prescribing in the context of antimicrobial resistance, and (v) laboratory testing and test results.

**Conclusions:**

A significant proportion of junior doctors report lone prescribing of antimicrobials in the context of low self-perceived confidence and knowledge in this field, and frequent difficulty in accessing help when necessary. Innovative training, targeting five specific areas identified through this needs assessment, is urgently needed by junior doctors practising in secondary care.

## Background

Appropriate antimicrobial prescribing is essential for optimal clinical care, patient safety, mitigation of antimicrobial resistance (AMR) [1], and reduction of healthcare associated infections [2]. However, up to 50 % of antimicrobial usage is reported to be suboptimal in acute care settings [3]. Improving healthcare professionals’ education has recently been widely promoted as a method for potentially encouraging more appropriate use of antimicrobials and improving clinical practice [4–6]. Such education is an essential component of antimicrobial stewardship programmes [7] and a national self-assessment toolkit for organisations, designed to assess their antimicrobial stewardship programmes, recognises education and training of prescribers as an integral component of the organisational approach [8]. Similarly, a recent consensus on reducing medication errors recommended provision of sufficient training of medical students and newly qualified doctors to ensure safer prescribing [9, 10].

Although it is recognised that knowledge and experience are required to optimally prescribe antimicrobials, prescribing decisions are often left to junior doctors [11, 12]. These newly qualified clinicians are a large prescribing group and the most mobile workforce within the National Health Service (NHS) in the United Kingdom (UK), as bi- or tri-annual rotations often result in movement between hospital groups (i.e. Trusts). However, junior doctors, particularly those just starting to practice, may not have the expertise, knowledge or confidence to optimally prescribe antimicrobials, and seniors may not always have the opportunity to review prescriptions written by the juniors working with them [13]. Although junior doctors admit that antimicrobial prescribing is a challenging and complex task, especially for those who are at the beginning of their training [14], they tend to underestimate their own responsibility for preventing AMR [15–17].

Whilst previous exploratory studies have looked at the issues around antimicrobial prescribing mainly for medical students (who are not yet prescribers), including in the United States [18], Europe [19–21] and Democratic Republic of the Congo [22], many of these issues are context specific. UK junior doctors’ needs and understanding in AMR and antimicrobial stewardship must be explored if interventions to improve prescribing are to be effective. As not all educational methods are appropriate or successful for adult learners, it is also important to involve junior doctors as co-designers of future educational strategies [14].

This study aims to identify current self-perceived gaps in junior doctors’ knowledge, and to understand their perceptions, regarding antimicrobial prescribing. Obtaining a clear picture of this will enable (i) targeted educational programmes to be developed for junior doctor continuing professional development, (ii) inform revision of post-graduate curricula in the area of antimicrobial prescribing and stewardship, and (ii) set a benchmark against which the efficacy of interventions such as these can be assessed.

## Methods

### Design and setting

A cross-sectional survey of junior doctors in post-graduate training posts in a multicentre teaching hospital network in London, UK, was undertaken in April 2014. The hospital network comprises five hospitals on four sites providing approximately 1500 inpatient beds and nine satellite clinics. To support appropriate antimicrobial prescribing, there is an active antimicrobial stewardship program in place for all hospitals in the network delivered through a multidisciplinary integrated team, i.e. pharmacists, infection control practitioners, and microbiology/ infectious disease physicians.

### Participants and recruitment

All junior doctors (i.e. post-qualification from medical school yet who are still in post-graduate specialty training) at the host hospital network were invited to take part in the study. This included the first 2 years post-qualification (in the UK Foundation Year (FY) 1 and FY2 otherwise known as internship) and three to eight years post-qualification (in the UK core trainees (CT), specialty trainees (ST), and specialist registrars (SpRs), otherwise known as residency). The first 2 years of training involve a general approach of learning the broad spectrum of the medical and surgical curriculum, whereas the 3^nd^ year and plus will have an additional specialty to learn in depth.

Recruitment involved both active participant invitations at 16 post graduate teaching sessions in three different hospitals and dissemination of an electronic survey to all junior doctors in post in April 2014 via their hospital network email accounts. The decision to use both methods was made prior to the start of the study. The post graduate teaching sessions are weekly mandatory teaching sessions for all junior doctors, who are expected to attend 70 % of these sessions over an academic year. They cover the abridged post graduate curriculum, without being infection specific, and are part of the continuous professional development for doctors. Direct recruitment at junior doctor training events continued until saturation was reached, as defined by 85 % or more of doctors in training in a session reporting that they had completed the survey already. In order to enhance participation from more senior grade junior doctors, the questionnaire was circulated by an embedded link in an invitation email. A reminder email was sent to all the participants at 2 weeks. A tracking number was generated for each participant to ensure confidentiality. All participants were eligible to enter in a prize draw for one of twenty-five £25 ($37USD) gift vouchers.

### Data collection

Participants were invited to complete a 45-item questionnaire on antimicrobial prescribing practices, previous education including medical degree and post-degree training, learning interests, and demographics, that lasted approximately 10 min. The questionnaire had been piloted by 6 healthcare professionals, including 3 infectious disease doctors, in order to assess the clarity and the length of the questions. The questions were constructed following a comprehensive literature review. With respect to antibiotics, participants were asked about prescribing practice; desire for additional training; confidence in prescribing; attitudes toward prescribing policies, healthcare associated infections and AMR; knowledge of prescribing policy and AMR; influences on prescribing practice; sources of information used for prescribing; as well as desirable topics to receive training on and the type and format for such training. All questionnaires were completed anonymously to increase reporting of sensitive information.

The electronic questionnaire was identical to the paper-based one, but delivered via Adobe® FormsCentral. A protocol for data entry was developed and training was provided to ensure consistency between researchers. Information derived from paper-based questionnaires was double-entered into a Microsoft® Access database for accuracy and all inconsistencies were investigated and resolved. Information derived from Adobe Forms was automatically exported to Microsoft Excel.

### Data analysis

Associations between demographics, training interests and attitudes and knowledge to antibiotic prescribing were explored, as was confidence in prescribing and demographics, education history, and year in training by cross tabulations, tests of central tendency and stepwise multivariate logistic regression using a backward elimination approach. All the variables of interest were entered in the multivariate analysis. The reported p-values were considered as two-tailed, and a p-value <0.05 was considered to be significant. Statistical analysis was performed using STATA version 12 (STATA Corp, College Station, TX).

## Results

Among 130 junior doctors actively approached during teaching sessions, 109 (response rate 84 %) completed the paper-based questionnaire. These sessions were mainly attended by 1^st^ and 2^nd^ year post-qualified doctors. The survey was sent electronically to 759 junior doctors who were registered with North West London region; a total of 31 completed the questionnaire (response rate: 4 %); however not all of those on the email distribution list would have been posted to the host Trust during the April 2014 period, and therefore have had access to their hospital email. Of the total of 140 respondents, 75 (54 %) were female, 109 (80 %) were under 30 years-old and 103 (74 %) were in their 1^st^ or 2^nd^ post-qualification years (Table 1).

**Table 1:** Characteristics of Junior Doctors enrolled in the study (Health Education North West London, April 2014)

N total participants = 140	N^a^ (%)
Gender	
Male	63 (45.7 %)
Female	75 (54.3 %)
Age (years)	
22–25	57 (41.6 %)
26–29	52 (38.0 %)
30+	28 (20.4 %)
Current post	
1^st^ year post-qualified	58 (41.5 %)
2^nd^ year post-qualified	45 (32.1 %)
≥ 3^rd^ year post-qualified	37 (26.4 %)
Country of medical training	
UK	129 (94.2 %)
Outside of UK	8 (5.8 %)
First post-qualified post	
Medicine	80 (58.8 %)
Surgery	54 (39.7 %)
Other	2 (1.5 %)
Currently prescribing antimicrobials in their post	
Yes	134 (95.7 %)
No	6 (4.3 %)

^a^Presence of missing values if the total of answers per category does not equal 140

### Prescribing behaviour

**Fig. 1:**
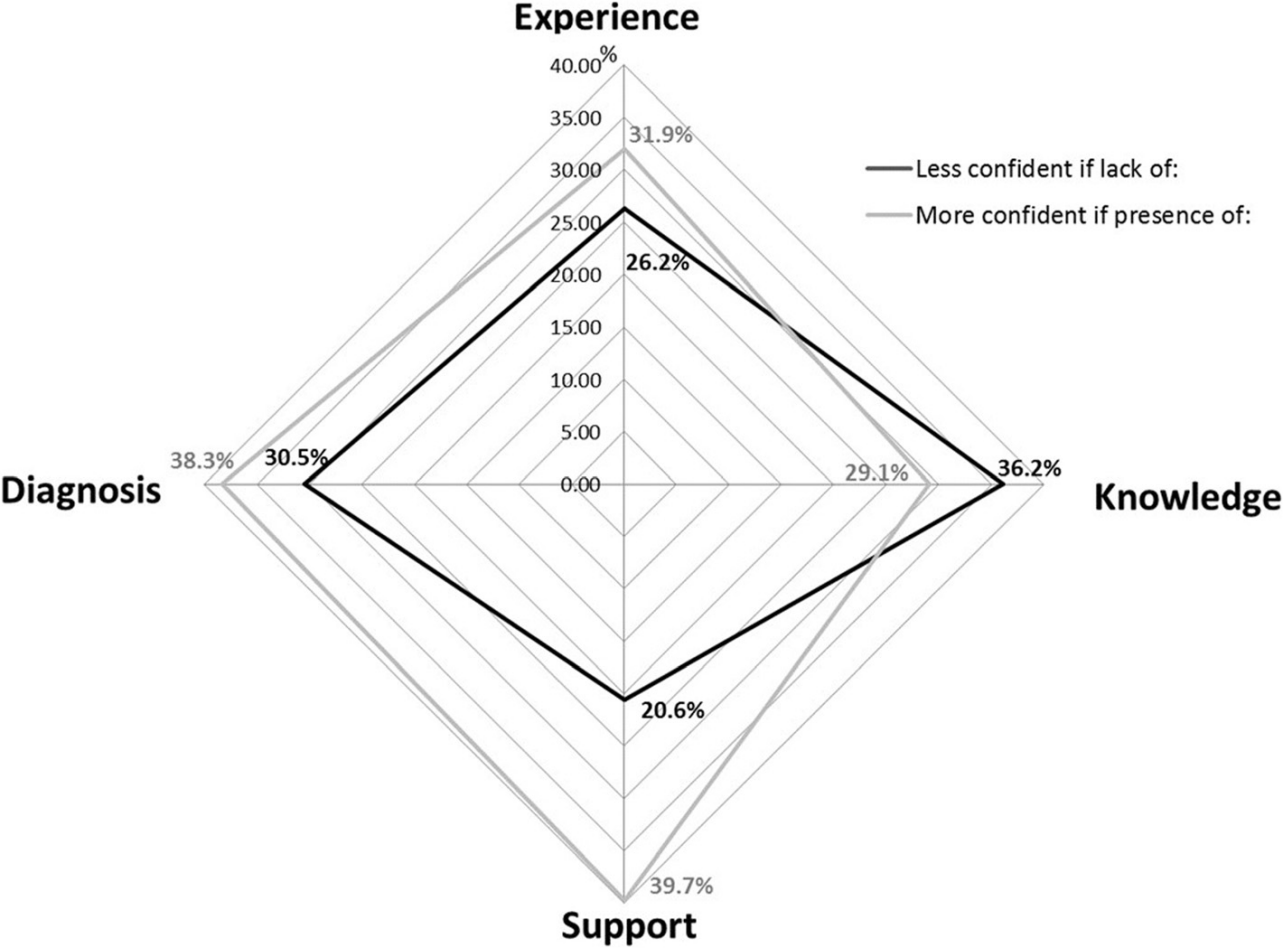
Factors influencing junior doctor confidence around antimicrobial prescribing (*n* = 140). Legend: This figure represents each of the 4 factors reported as influencing antimicrobial prescribing confidence by junior doctors. These factors form individual axes which have been arranged radially around a point. The value of each aspect is depicted by the node (anchor) on the spoke (axis). A line is drawn connecting the data values for each spoke. Percentages represent the proportions of respondents stating the variable influencing their confidence

Whilst junior doctors in their first year post-qualification rarely (*n* = 7, 13 %) reported prescribing primarily without senior supervision, those with just 1 year more experience reported doing so frequently (*n* = 18, 46 %). Junior doctors also reported feeling increased confidence in prescribing in this 2^nd^ year post-qualification (*n* = 34, 92 %) compared to their 1^st^ (*n* = 36, 64 %). However whilst both doctors who were in their 2^nd^ or ≥3^rd^ year post-qualification reported feeling increased confidence in antimicrobial prescribing, they were also more likely to report a need for further antimicrobial education (respectively, *n* = 32, 74 and *n* = 29, 74 %) than those in their 1^st^ year post-qualification (*n* = 35, 60 %). Reported factors influencing confidence in antimicrobial prescribing (Fig. 1) were that a lack of knowledge decreased confidence (36 %), but conversely the presence of knowledge did not necessarily improve confidence. Instead appropriate support (40 %) and diagnosis confirmation (39 %) were reported as key factors to improving confidence.

When asked about two key antimicrobial prescribing behaviours, that of considering AMR, and that of de-escalation of prescriptions, variation was evident between levels of respondent experience. First, appreciation of AMR as a prescription-altering factor was more prevalent among those in their later years of practice (*n* = 45 80, *n* = 29 88, and *n* = 13 100 % for 1^st^, 2^nd^ and ≥3^rd^ year post-qualified, respectively). Second, for prescription de-escalation in line with national policy [23], 1^st^ and ≥3^rd^ year post-qualified doctors reported concording with policy guidelines only infrequently (respectively *n* = 12, 22 and *n* = 6, 18 %), but those in their 2^nd^ year-post-qualification reported observing this guidance in over half of all cases (*n* = 20, 53 %). Only a small proportion of doctors in the three groups believed that non-optimal (0–23 %), or unsafe (14–35 %), antimicrobial prescriptions are currently reported back to prescribers to enable learning from mistakes (Table 2).

**Table 2:** Comparison of the prescribing practices, needs and knowledge between post-qualification juniors doctors in London (*n* = 140^a^)

	1^st^ year post-qualified n (%) (*N* = 58)	2^nd^ year post-qualified n (%) (*N* = 45)	≥3^rd^ year post-qualified n (%) (*N* = 37)	*P* value^b^
Prescribing practice				
How often do you prescribe antimicrobials?^c^				
≤ once a week	3 (5.4)	8 (21.6)	6 (16.7)	
2–4 times/week	28 (50.0)	14 (37.8)	16 (44.4)	
≥ 1/day	25 (44.6)	15 (40.6)	14 (38.9)	0.21
Do you prescribe with a senior doctor?^c^				
Primarily without senior supervision	7 (12.5)	18 (46.2)	20 (57.1)	
Sometimes with a senior doctor	23 (41.1)	10 (25.6)	11 (31.4)	
More often with a senior doctor	26 (46.4)	11 (28.2)	4 (11.5)	<0.01
If a non-optimal antimicrobial prescription is noticed, would it be reported back to the prescriber?				
Yes, all the time	0	1 (3.0)	6 (23.1)	
sometimes	18 (46.2)	21 (63.7)	11 (42.3)	
Rarely	17 (43.6)	10 (30.3)	5 (19.2)	
Never	4 (10.2)	1 (3.0)	4 (15.4)	<0.01
If an unsafe antimicrobial prescription is noticed, would it be reported back to the prescriber?				
Yes, all the time	6 (14.0)	12 (35.3)	5 (21.7)	
sometimes	24 (55.8)	19 (55.9)	14 (60.9)	
Rarely	12 (27.9)	3 (8.8)	2 (8.7)	
Never	1 (2.3)	0	2 (8.7)	0.05
Do you consider AMR when prescribing?				
Yes	45 (80.4)	29 (87.9)	13 (100.0)	
No	11 (19.6)	4 (12.1)	0	0.24
How often do you consider IV to oral switch?				
Every 24 h	12 (21.8)	20 (52.6)	6 (17.6)	
> 24 h	13 (23.6)	2 (5.3)	7 (20.6)	
Different case by case	30 (54.6)	16 (42.1)	21 (61.8)	<0.01
Do you find easy to switch IV to oral? ^c^				
Yes	9 (16.4)	11 (29.0)	16 (47.1)	
No	14 (25.4)	7 (18.4)	6 (17.6)	
Sometimes	32 (58.2)	20 (52.6)	12 (35.3)	0.04
Perception about training on antimicrobial prescribing				
Do you feel confident about antimicrobial prescribing?				
No	20 (35.7)	3 (8.1)	3 (8.1)	
Yes	36 (64.3)	34 (91.9)	34 (91.9)	<0.01
What is your current most effective training?				
Prescribing alone on the job	4 (7.4)	4 (9.3)	4 (10.2)	
Prescribing with seniors on the job	18 (33.3)	15 (34.9)	6 (15.4)	
Ward rounds	3 (5. 6)	4 (9.3)	7 (18.0)	
Teaching sessions	4 (7.4)	2 (4.6)	5 (12.8)	
Reading policy/ Self-study	25 (46.3)	18 (41.9)	17 (43.6)	0.34
From whom did you learn the most?^c^				
Doctors in my specialty training	14 (25.00)	22 (51.2)	12 (33.3)	
Consultants	2 (3.6)	4 (9.3)	4 (11.1)	
Infection specialists/ microbiologists	22 (39.3)	13 (30.2)	16 (44.5)	
Pharmacists	18 (32.1)	4 (9.3)	4 (11.1)	<0.01
Would you like more training in antimicrobial prescribing?^c^				
Yes	35 (60.3)	32 (74.4)	29 (74.4)	
No	19 (32.8)	9 (20.9)	8 (20.5)	
I do not know	4 (6.9)	2 (4.7)	2 (5.1)	0.55

^a^Presence of missing values if the total of answers per category does not equal 140

^b^Statistical significance are by Fisher exact test and Chi2 Test based on p value <0.05

^c^Variables tested in the multivariate model examining the factors associated with confidence prescribing antimicrobials as a junior doctor

### Prescribing support

Whilst junior doctors in their 2^nd^ year post-qualification indicated that within-specialty seniors were most often their key educators and role models for antimicrobial prescribing (*n* = 22, 51 %), among 1^st^ and ≥3^rd^ year post-qualified respondents infection specialists/microbiologists represented the most frequently cited sources of influence and education (respectively *n* = 22, 39 and *n* = 16, 45 %) (Table 2). Despite this expressed influence from seniors and specialists, and the impact on prescribing confidence provided by appropriate support noted above, around half of the doctors reported difficulty obtaining support on weekends (52) and at night (45 %).

### Prescribing education

**Fig. 2:**
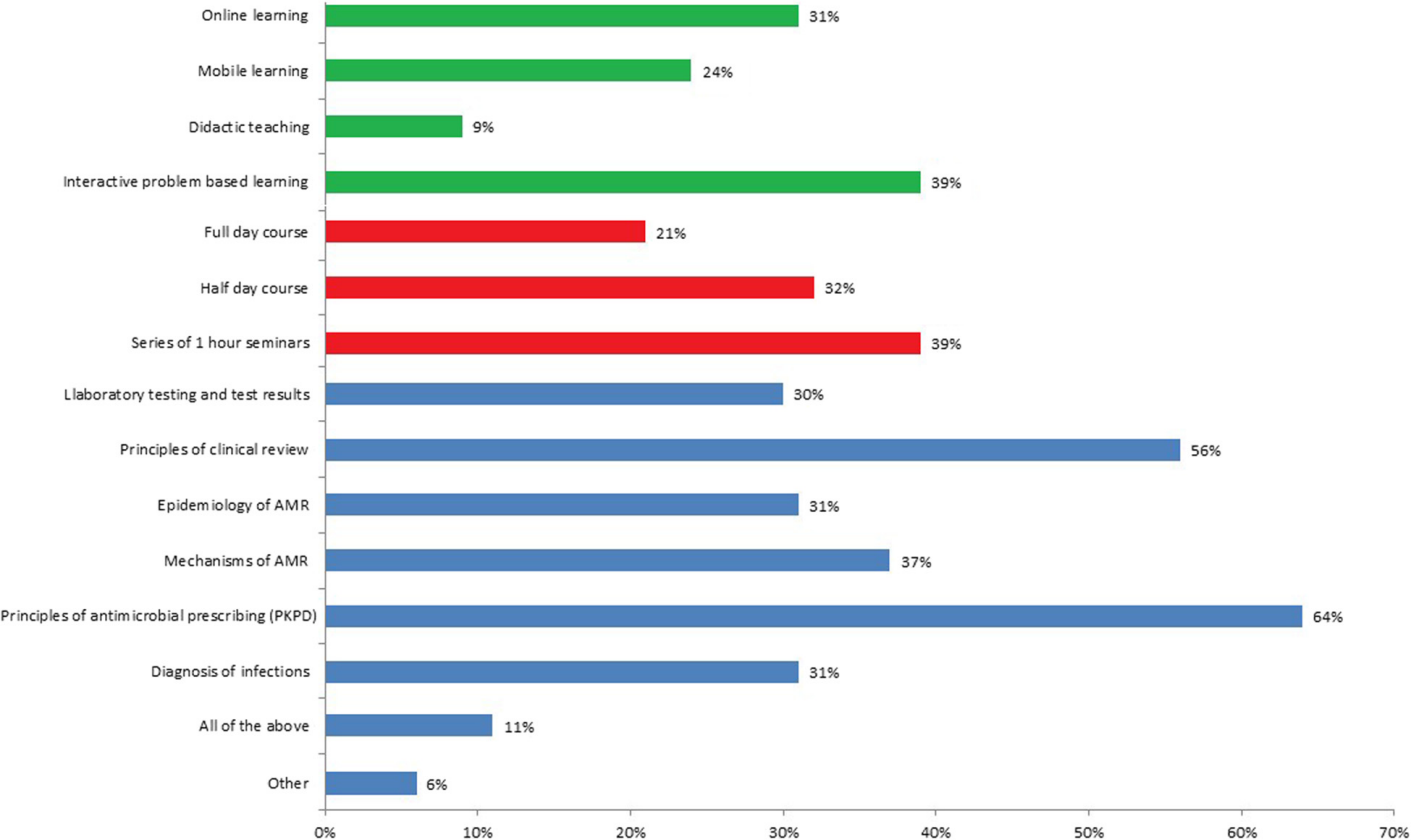
Characteristics of additional antimicrobial prescribing training that junior doctors would like to receive (*n* = 140). Legend: Proportion of respondents indicating a preference for type of education delivery (green), format of education (red) and content of educational activity (blue)

Across all respondents, irrespective of their number of years post-qualification, only a small percentage of participants found current teaching sessions to be effective (5–13 %), whilst a large proportion (42–46 %) reported learning better through self-education and reading policies (Table 2). Respondents indicated that they would like additional training to be delivered via Problem-Based Learning (39 %) in the context of series of one hour seminars (39 %) or half day courses (32 %) (Fig. 2). Respondents suggested that the content of the course should mainly cover the following themes: (i) principles of antimicrobial prescribing (64 %), (ii) diagnosis of infections (31 %), (iii) clinical review of patients with infections (57 %), (iv) aspects of antimicrobial resistance (37 % reported wanted teaching on mechanisms of resistance, 31 % on epidemiology), and (v) the role of laboratory testing and test results in prescribing (30 %) (Fig. 2).

### Multiple logistic regression analysis

Investigating the factors impacting junior doctors confidence in prescribing antimicrobials (Table 3), men were significantly more likely to report being confident than women (Odds Ratio [OR] =2.52 (Confidence Interval [CI], 1.00–6.55)) and both age groups 26–29 years-old and ≥30 years-old reported more confidence than the 22–25 years-old group in the univariate analysis (respectively, OR = 3.17 [CI, 1.13–8.93] and OR = 3.03 [CI, 0.79–11.61]) but not in the multivariate analysis. After adjusting for all potential confounders in the multiple logistic regression model, junior doctors’ reported confidence in prescribing antimicrobials was greater among those with more experience, i.e. their number of years in practice (OR = 6.97 [CI, 1.25–38.98] for 2^nd^ year post-qualified and OR = 5.43 [CI, 1.01–29.17] for ≥3^rd^ year post-qualified versus 1^st^ year post-qualified) and the frequency with which they reported currently prescribing antimicrobials (OR = 9.28 (CI, 1.32–65.15) when prescribing 2–4 times a week versus less than once a week). Junior doctors who reported prescribing primarily without senior supervision (OR = 10.97 [CI, 1.02–117.71] versus those who indicated that they mostly prescribed with a more senior doctor), as well as those who found the switch from intravenous to oral easy (OR = 11.66 (CI, 1.59–85.56) versus those who found it more difficult) reported increased confidence in prescribing. Yet, confidence was lower for those who wanted more training in antimicrobial prescribing (OR = 0.15 [CI, 0.03–0.69]).

**Table 3:** Multiple Logistic regression examining associated factors with confidence prescribing antimicrobials as a junior doctor (*n* = 140)

Associated factors	Unadjusted OR	[95 % CI]	Crude p-value^b^	Adjusted OR	[95 % CI]	Adjusted p-value^b^
Gender						
Female	1^a^					
Male	2.52	[1.00–6.55]	0.05			
Age (year)						
22–25	1^a^					
26–29	3.17	[1.13–8.93]	0.03			
30+	3.03	[0.79–11.61]	0.11			
Stage of medical training						
1^st^ year post-qualified	1^a^			1^a^		
2^nd^ year post-qualified	6.30	[1.71–23.12]	<0.01	6.97	[1.25–38.98]	0.03
≥ 3^rd^ year post-qualified	6.30	[1.71–23.12]	<0.01	5.43	[1.01–29.17]	0.05
Medical degree training						
4 years graduate course	1^a^					
5 years undergraduate entry	1.91	[0.52- 6.99]	0.33			
6 years undergraduate entry	1.48	[0.36–6.20]	0.59			
Frequency of antimicrobial prescribing						
≤ once a week	1^a^			1^a^		
2–4 times/week	2.04	[0.59–7.09]	0.26	9.28	[1.32–65.15]	0.02
≥ 1/day	1.63	[0.47–5.60]	0.44	5.24	[0.87–31.68]	0.07
Prescribing alone or not						
Mostly with a more senior doctor	1^a^			1^a^		
Sometimes with a more senior doctor	0.76	[0.30–1.94]	0.57	0.56	[0.17–1.80]	0.33
Primarily without senior supervision	15.61	[1.92–127.25]	0.01	10.97	[1.02–117.71]	0.05
To find easy to decide to de-escalate						
No	1^a^			1^a^		
Yes	8.05	[1.57–41.17]	0.01	11.66	[1.59–85.56]	0.02
Sometimes	1.69	[0.63–4.55]	0.30	3.40	[0.89–12.98]	0.07
From whom they learnt the most about antimicrobial prescribing						
Doctors in my specialty training	1^a^					
Consultants	1.47	[0.16–13.70]	0.73			
Infection specialists/ microbiologists	0.88	[0.29–2.65]	0.81			
Pharmacists	0.39	[0.12–1.25]	0.11			
Want more training						
No	1^a^			1^a^		
Yes	0.32	[0.09–1.15]	0.08	0.15	[0.03–0.69]	0.01
Don’t know	0.16	[0.02–1.00]	0.05	0.11	[0.01–1.14]	0.06

^a^Reference

^b^Statistical significance is based on p value <0.05

## Discussion

Our findings showed that a high proportion of junior doctors (13 %–57 %) reported prescribing antimicrobials without senior supervision, even during their first year of training post-qualification, yet 36 % of respondents self-report low confidence in their ability to complete this task. Respondents cited lack of knowledge as a key reason for this, and going forward the specific topics identified in this study will enable targeted educational programmes and revision of post-graduate curricula to optimise antimicrobial prescribing and stewardship. Yet we also found that increasing knowledge as an isolated variable may not necessarily reciprocally increase confidence; greater support (from seniors and specialists) and more certainty in the diagnosis of infection were stated to drive prescribing confidence. However, junior doctors across the study hospitals noted difficulty in accessing help when necessary, not only during nights and week-ends but also a surprising minority during standard working hours (8 %). Whilst it is essential to improve antimicrobial prescribing knowledge, structural and organisational changes must be enacted in parallel, including through decision support tools, and improved diagnostic tests, to enable junior doctors to gain confidence in this field. Similarly, the perception of junior doctors that feedback in cases of sub-optimal, or even unsafe, antimicrobial prescribing is infrequent and unreliable, raises concern. Feedback mechanisms to support quality improvement and patient safety are being developed in healthcare settings addressing a variety of service issues related to this [24, 25]. However, mechanisms to report antimicrobial prescribing issues back to the prescribers are not sufficient and must be enhanced, increasing guideline concordance, improving knowledge, and engendering best practice among junior doctors.

Whilst we found that junior doctors reported co-prescribing with a senior less frequently as they progressed in experience, co-prescribing still occurred for 43 % of those who had been qualified for ≥3 years. Furthermore, beyond simply co-prescribing, junior doctors also report numerous sources of support for their prescribing activities. In fact, junior doctors reported that their seniors were one of the most influential actors on their antimicrobial prescribing practice; for those in their second year post-qualification, seniors were more influential even than infection specialists, perhaps because of comparative frequency of contact. This finding correlates with previous work showing the importance of the professional hierarchy and the existence of “prescribing etiquette” as a determinant of antimicrobial prescribing [12]. Therefore, one should consider whether education aimed to optimise antimicrobial prescribing would be most effective among junior doctors, or should perhaps also target seniors. We also acknowledge that further research on more senior level should be conducted. We suggest however, that given we found that a lack of knowledge was associated with low confidence, focussed training (mindful of structural and organisation changes) is likely to increase competence and confidence and enable juniors doctors to challenge existing hierarchies and promote good practice. However, improving knowledge should be supplemented with enhanced decision making skills, as well as communication and negotiation skills in order to impact “prescribing etiquette”. In the context of a multi-modal approach to antimicrobial stewardship, the data supports an essential need to improve access to infection specialists, and to put them at the centre of antimicrobial prescribing education.

Given the need for education on antimicrobial prescribing among junior doctors, their perceived needs in terms of content and delivery were also evident from our data. First, up to 20 % of junior doctors, mainly 1^st^ year post-qualification, did not take into consideration AMR when prescribing antimicrobials; such awareness only becomes prevalent in later years, indicating a need for targeted education on the practical implications of AMR early in post-graduate education. Of note, whilst 20 % of prescribers declared that they do not consider AMR when prescribing, there is perhaps cause for optimism given comparator data on appreciation of AMR in prescribing from previous studies [15, 26]. Second, one of the key antimicrobial stewardship principles - “Start Smart and then Focus” [23] - (which promotes the review of the prescriptions every 24 h with de-escalation from intravenous to oral when possible), is practiced twice as frequently by the 2^nd^ year post-qualified junior doctors than 1^st^ or ≥3^rd^ years. This suggests that key components of antimicrobial stewardship programmes, such as “Start Smart Then Focus” need to be highlighted early in post graduate medical education, but then must be reinforced in later years when more experienced junior doctors have other competing considerations. Third, we found that junior doctors self-reported a need for additional training in the areas of both clinical review of infected patients, and principles of prescribing. This links to established patient safety agendas, and clearly establishes a need for education on sepsis resuscitation [27], and therapeutic drug monitoring [28, 29] respectively.

The identified need for further infection education must be catered for through a learner-centred, mixed method approach and such educational interventions must have a mechanism for evaluating their efficacy. Our data suggests passive educational activities, such as didactic teaching sessions, are not of interest to junior doctors. Rather, interactive approaches such as problem based learning delivered in either one-hour seminars or a half day course are called for, as are learning mechanisms accessible through mobile and on-line platforms; findings compatible with schedules of full-time working professionals, and in line with previous studies [30–33].

The findings from this study have several limitations. First, the sample predominantly captured the most junior doctors (74 % were 1^st^ or 2^nd^ year post-qualified). We do not know what proportion of prescriptions is made by this group in contrast to those in later years of training. Our results showed that there were no significant differences between the three groups in terms of antimicrobial prescribing frequency. However, further research needs to be conducted on more senior doctors (trainees and consultants) who have limited time for training. Second, our participation rate was excellent for our paper-based survey involving active recruitment during teaching sessions (84 %) but poor for the electronic version sent via email. This may explain the low participation rate among junior doctors ≥3 years qualified. We may have captured those with more interest in the subject and therefore more knowledge or confidence in prescribing antibiotics. Third, our study has been limited to a London hospital network where the culture of antimicrobial stewardship is reasonably ensconced across the multi-professional healthcare team, possibly influencing responses [34, 35]. However, the participating junior doctors had received their undergraduate medical education from numerous medical schools across the UK, with fairly standardised curricula in the field of AMR [36], suggesting that our results may be generalisable across the UK, but less likely to other countries where the curriculum on this topic may differ significantly. Lastly, our study described the self-reported perceptions and behaviour of junior doctors’ antimicrobial prescribing practice. An observational study objectively assessing knowledge and behaviour around antimicrobial prescribing is clearly indicated.

## Conclusion

This study highlights the need for focused, learner-centred, mixed method approaches to antimicrobial prescribing education among junior doctors. Moreover for the first time specific self-identified learning needs have been identified for this to occur, enabling organisations to create targeted educational programmes and revise post-graduate curricula to optimise antimicrobial prescribing and stewardship. However it also underlines the need for education to be ensconced within an organisational structure providing appropriate infection specialist, decision making, and diagnostic support. To meet these needs, the findings from this study have informed the ongoing development of an educational tool (a Continuing Professional Development accredited short course) which is being validated by junior doctors. This educational tool also uses online and mobile learning that interactively delivers knowledge and will hopefully shape behaviours and attitudes in the areas of (i) principles of antimicrobial prescribing, (ii) diagnosis of infections, (iii) clinical review of patients with infections, (iv) prescribing in the context of antimicrobial resistance, and (v) the role of laboratory testing and test results in prescribing.
